# Combination Therapy Using Chimeric Monoclonal Antibodies Protects Mice from Lethal H5N1 Infection and Prevents Formation of Escape Mutants

**DOI:** 10.1371/journal.pone.0005672

**Published:** 2009-05-22

**Authors:** Mookkan Prabakaran, Nayana Prabhu, Fang He, Qian Hongliang, Hui-Ting Ho, Jia Qiang, TaoMeng, Michael Goutama, Jimmy Kwang

**Affiliations:** 1 Animal Health Biotechnology, Temasek Life Sciences Laboratory, National University of Singapore, Singapore, Singapore; 2 Tridel Biosciences International Pte Ltd, Singapore, Singapore; 3 Department of Microbiology, Faculty of Medicine, National University of Singapore, Singapore, Singapore; Hallym University, Republic of Korea

## Abstract

**Background:**

Given that there is a possibility of a human H5N1 pandemic and the fact that the recent H5N1 viruses are resistant to the anti-viral drugs, newer strategies for effective therapy are warranted. Previous studies show that single mAbs in immune prophylaxis can be protective against H5N1 infection. But a single mAb may not be effective in neutralization of a broad range of different strains of H5N1 and control of potential neutralization escape mutants.

**Methods/Principal Findings:**

We selected two mAbs which recognized different epitopes on the hemagglutinin molecule. These two mAbs could each neutralize in vitro escape mutants to the other and in combination could effectively neutralize viruses from clades 0, 1, 2.1, 2.2, 2.3, 4, 7 and 8 of influenza A H5N1 viruses. This combination of chimeric mAbs when administered passively, pre or post challenge with 10 MLD50 (50% mouse lethal dose) HPAI H5N1 influenza A viruses could protect 100% of the mice from two different clades of viruses (clades 1 and 2.1). We also tested the efficacy of a single dose of the combination of mAbs versus two doses. Two doses of the combination therapy not only affected early clearance of the virus from the lung but could completely prevent lung pathology of the H5N1 infected mice. No escape variants were detected after therapy.

**Conclusions/Significance:**

Our studies provide proof of concept that the synergistic action of two or more mAbs in combination is required for preventing the generation of escape mutants and also to enhance the therapeutic efficacy of passive therapy against H5N1 infection. Combination therapy may allow for a lower dose of antibody to be administered for passive therapy of influenza infection and hence can be made available at reduced economic costs during an outbreak.

## Introduction

The recent emergence of H5N1 strains of influenza A virus and the high mortality caused by them in humans has raised concerns for the possibility of a future influenza pandemic. Present vaccine strategies have been hindered by antigenic variation of the influenza strains [1]. Vaccine strategies requiring endogenous synthesis of antibodies will not provide the immediate protection needed against H5N1 infections in the event of a pandemic. Antiviral therapy has received much attention during these situations. However, currently available anti-viral treatment options are limited [2]. Isolation of drug-resistant viral strains [3], [4] in the recent past warrants an urgent need for alternative strategies for treatment and prophylaxis. Passive administration of antibodies against neutralizing epitopes of H5N1 may be an attractive alternative to active vaccination of humans, in particular for those individuals who are at high risk from influenza infection, viz. the immuno-compromised patients or the elderly who do not respond well to active immunization [5].

Antibody based therapy is one of the alternative approaches for the immunoprophylaxis or the treatment of influenza and other infections. Passive administration of polyclonal antibodies against H5N1 has been shown to be protective in several non-primate and human models of infection [6]. Passive immunization by transfusion of human convalescent sera was associated with 50% reduction in mortality during an influenza pandemic and was shown to be effective against H5N1 influenza A viral infection [6], [7]. Equine F (ab') 2 fragments specific for H5N1 have been used for efficacious prophylaxis and therapy in a mouse model [8]. Murine monoclonal antibodies (mAbs) against fusion peptide of hemagglutinin (HA) of H5N1 influenza have been shown in passive transfer experiments to protect mice from infection by reduction of viral replication [9]. Thus passive administration of mAbs prior to or after influenza infection has the potential advantage of providing high titers of antibodies to susceptible individuals immediately. Murine mAbs were used in initial clinical trials. The efficacy of these mAbs was hampered by several problems including their diminished serum half-life and the development of human anti-mouse antibodies (HAMA) [10]. To counter this problem, several strategies have been devised including the generation of chimeric, humanized and human mAbs.

Currently, there has been a lot of focus on therapeutic approaches using neutralizing antibodies against the HA1 protein of the influenza virus. This protein is easy to target as it is on the surface of the virus and antibodies against this protein can neutralize the virus efficiently. MAb prophylaxis, targeting the HA protein, may be an effective means of controlling an influenza outbreak. Passive immunoprophylaxis and therapy with a single neutralizing humanized or human mAb was efficacious against lethal challenge with specific strains of H5N1 virus [11], [12]. It is important that any mAb product should offer broad protection against all circulating strains of H5N1 influenza and should prevent the selection of neutralization escape mutants *in vivo*. A single monoclonal antibody may not be efficient in meeting the above criteria.

Several factors are important in forming an effective combination of mAbs in the prophylactic and therapeutic regimen against H5N1 influenza infection. These include inclusion of an ideal pair of complementing monoclonal antibodies, optimizing the number of doses, time intervals in between doses and the duration of therapy. The mAbs included in the combination therapy should target distinct regions on the antigen with non overlapping epitopes and should be able to complement each other in a treatment regimen [13], [14]. In the present study, we focus on the selection of a pair of mAbs against two different neutralizing epitopes of H5N1 and their chimerization. We evaluate the prophylactic and therapeutic efficacy of the combination therapy using the chimeric mAbs in a murine model, experimentally challenged with two distinct phylogenetic clades of highly pathogenic H5N1 viruses.

## Materials and Methods

### Viruses

H5N1 human influenza viruses from clade 2.1 A/Indonesia/CDC669/2006, A/Indonesia/TLL013/2006, A/Indonesia/CDC540/2006, A/Indonesia/CDC594/2006 and one avian strain A/Indonesia/TLL014/2006 were obtained from the Ministry of Health (MOH), Republic of Indonesia. The other subtypes of influenza A viruses, H3N2 (A/chicken/Singapore/Sin/92) and H7N1 (A/common iora/Indonesia/F89/11/95) were obtained from the Agri-Food and Veterinary Authority (AVA) of Singapore. The H5N1 viruses from different phylogenetic clades/subclades were rescued by Reverse Genetics [15]. Briefly, the hemagglutinin (HA) and neuraminidase (NA) genes of H5N1 viruses from clades 0, 1, 2.1, 2.2, 2.3, 4, 7 and 8 (Table 1) were synthesized (GenScript, USA) based on the sequence from the NCBI influenza Database. The synthetic HA and NA genes were cloned into a dual-promoter plasmid for influenza A reverse genetics [15]. The dual-promoter plasmids were obtained from Center for Disease Control and Prevention, Atlanta, GA, USA. Reassortant viruses were rescued by transfecting plasmids containing HA and NA along with the remaining six influenza genes derived from A/Puerto Rico/8/34 (H1N1) into co-cultured 293T and MDCK cells using Lipofectamine 2000 (Invitrogen Corp.). At 72 h post-transfection the culture medium was inoculated into embryonated eggs or MDCK cells. The HA and NA genes of reassortant viruses from the second passage were sequenced to confirm presence of introduced HA and NA genes and the absence of mutations. Stock viruses were propagated in the allantoic cavity of 11 day-old embryonated eggs [16], virus containing allantoic fluid was harvested and stored in aliquots at −80°C. Virus content was determined by standard hemagglutination (HA) assay [17]. All experiments with highly pathogenic viruses were conducted in a biosafety level 3 (BSL-3) containment facility in compliance with CDC/NIH and WHO recommendations [18], [19].

**Table 1:** Reassortant influenza A viruses generated by reverse genetics.

Serial No.	Virus name (subtype)#	Clade	Host
1	A/Hongkong/156/97 (H5N1)	0	Human
2	A/HongKong/213/03 (H5N1)	1	Human
3	A/Vietnam/1203/04 (H5N1)	1	Human
4	A/Indonesia/CDC1031/07 (H5N1)	2.1	Human
5	A/turkey/Turkey1/05 (H5N1)	2.2	Avian
6	A/barheaded goose/Qinghai/12/05(H5N1)	2.2	Avian
7	A/Nigeria/6e/07(H5N1)	2.2	Human
8	A/Egypt/0636-NAMRU3/07(H5N1)	2.2	Human
9	A/Anhui/1/05 (H5N1)	2.3	Human
10	A/chicken/Nongkhai/NIAH400802/07 (H5N1)	2.3	Avian
11	A/VietNam/HN31242/07 (H5N1)	2.3	Human
12	A/goose/Guiyang/337/06 (H5N1)	4	Avian
13	A/chicken/Shanxi/2/06 (H5N1)		
14	A/chicken/Henan/12/04 (H5N1)	8	Avian

# Donor of HA and NA genes for derivation of PR8 reassortant viruses.

### Production and characterization of mAbs

BALB/c mice were immunized twice subcutaneously at intervals of 2 weeks with purified formalin inactivated A/Indonesia/CDC669/2006 or A/Indonesia/TLL014/2006 antigen with adjuvant (SEPPIC, France). Mice were boosted with the same viral antigen, 3 days before the fusion of splenocytes with SP2/0 cells [20]. The fused cells were seeded in 96-well plates, and their supernatants were screened by immunofluorescence assays as described below. The hybridomas that produced the mAbs were cloned by limiting dilution at least three times. The positive mAbs were tested for their hemagglutination inhibition activity as described below. Immunoglobulins from selected positive mAbs were isotyped using a commercial isotyping kit (Amersham Bioscience, England) as described in the manufacturer's protocol.

### Immunofluorescence assay (IFA)

MDCK cells cultured in 96-well plates were infected with AIV H5N1 strains. At 24–48 h post-infection, the cells were fixed with 4% paraformaldehyde for 30 min at room temperature and washed thrice with phosphate buffered saline (PBS), pH 7.4. Fixed cells were incubated with hybridoma culture supernatant at 37°C for 1 h, rinsed with phosphate buffered saline (PBS) and then incubated with a 1∶40 dilution of fluorescein isothiocyanate (FITC)-conjugated rabbit anti-mouse Immunoglobulin (Dako, Denmark). Cells were rinsed again in PBS and antibody binding was evaluated by wide-field epi-fluorescence microscopy (Olympus IX71) [21].

### Hemagglutination inhibition assay

Hemagglutination inhibition (HI) assays were performed as described previously [22]. Briefly, mAbs were serially diluted (2 fold) in V-bottom 96-well plates and mixed with 4 HA units of virus (A/Indonesia/TLL013/06). Plates were incubated for 30 min at room temperature, and 1% chicken RBCs were added to each well. The hemagglutination inhibition endpoint was the highest mAb dilution in which agglutination was not observed.

### Isolation and analysis of escape mutants

The epitope recognized by mAb 2D9 and 4C2 were mapped by characterization of escape mutants as described previously [23]. Briefly, H5N1 viruses were incubated with an excess of mAb for 1 h and then inoculated into 11 day old embryonated chicken eggs. For isolation of *in vivo* escape mutants, the lung samples from the treated mice were inoculated directly into the embryonated eggs. The eggs were incubated at 37°C for 48 h. Virus was harvested and used for cloning in limiting dilution in embryonated chicken eggs and the escape mutants were plaque purified. The HA gene mutations were then identified by sequencing and comparing with the sequence of the parent virus.

### Cloning of chimeric IgG1 expression plasmid

Design of the expression vector was as described [24]. Briefly, human antibody constant regions encoding the kappa light chain and the IgG1 heavy chain were amplified and cloned into a modified pCMV/myc/ER plasmid with an internal ribosome entry site (IRES) of encephalomyocarditis virus inserted in between them. Unique restriction sites were introduced to allow for insertion of the variable regions of the heavy and light chains in frame with the constant regions.

mRNA was prepared from the mAb 4C2 and 2D9 hybridoma cells and used in first strand cDNA synthesis with random hexamers. The total cDNA was used as template to amplify both the variable heavy and light chain using the primers and protocols of the mouse scFv recombinant antibody phage system (Amersham Biosciences). The resultant products were cloned into pCR-Script (Stratagene, USA) for sequencing. Sequence-specific primers were then designed and used for amplification of the variable regions, which were then cloned into the expression vector. Expression of this construct lead to the production of chimeric antibodies containing 33% of the sequences as mouse variable regions from murine and 67% of the sequences as human constant regions for IgG1.

### Transient expression of chimeric antibodies and purification

Chimeric antibodies were expressed using the Freestyle 293 Expression system (Invitrogen, USA) to obtain antibodies produced in a defined, serum-free medium. The above mentioned construct was transfected into 293-F cells using 293fectin (Invitrogen, USA) and supernatants were collected 120 h after transfection. The chimeric antibodies 4C2 (ch-mAb 4C2) and 2D9 (ch-mAb 2D9) were purified using Protein A sepharose beads (Millipore). Purity of the chimeric antibodies were confirmed by SDS-PAGE and immunoblot analysis using HRP labeled anti-human Ig (DAKO) was used to confirm introduction of human constant regions.

### Microneutralization assay

Neutralization activity of the chimeric antibodies against H5N1 strains was analyzed by microneutralization assay as previously described [25], [26]. Briefly, mAb was serially two-fold diluted and incubated with 100 50% tissue culture infectious doses (TCID_50_) of different clades of H5N1 strains for 1 h at room temperature and plated in duplicate onto MDCK cells grown in a 96-well plate. The TCID_50_ of each of the H5N1 strains in MDCK cell culture was determined by the Reed and Muench method [27]. The neutralizing titer was assessed as the highest mAb dilution in which no cytopathic effect was observed by light microscopy.

### Immunization and Challenge

Groups of SPF female BALB/c mice aged 4–6 weeks were used for the challenge studies. Mice (n = 10 per group) were inoculated intranasally with 10 MLD_50_ (Mouse lethal dose 50%) of two different H5N1 strains (A/Vietnam/1203/2004 from clade 1 and A/Indonesia/TLL013/06 from clade 2.1). All animal experiments were carried out in accordance with the guides for animal experiments performed at NIID and experimental protocols were reviewed and approved by Institutional Animal Care and Use Committee at Temasek Life Sciences Laboratory, National University of Singapore.

### Prophylactic efficacy

To determine the prophylactic efficacy, mice were pre-treated intraperitoneally with 1.0 mg/kg, 2.5 mg/kg, 5 mg/kg or 0 mg/kg (PBS) of the combination of ch- mAbs, prior to the viral challenge. 5 mg/kg of an irrelevant IgG1 monoclonal antibody 8C2 (specific for porcine circovirus), prepared in a similar manner was used to measure any non-specific protection. After 24 h, mice were challenged with 10 MLD_50_ of the two different H5N1 strains. Mice were observed daily to monitor body weight and mortality until all animals died or until day 14 after challenge.

### Therapeutic efficacy

To determine the therapeutic efficacy of the ch-mAb, each group of mice was experimentally infected with 10 MLD_50_ of the two different H5N1 strains. Twenty four hours after viral infection, the mice were treated via intra-peritoneal route with 1.0 mg/kg, 2.5 mg/kg, 5 mg/kg or 0 mg/kg (PBS) of the combination of ch-mAbs. 5 mg/kg of an irrelevant monoclonal antibody 8C2 (specific for porcine circovirus) prepared in a similar manner was used to measure any non-specific protection. For the double therapy experiment, different sets of mice were treated with similar doses of chimeric mAbs 24 h and 72 h after the viral challenge.

One additional group of mice was challenged with 10 MLD_50_ of H5N1 virus from clade 2.1 and treated one day after viral challenge with ch-mAb 2D9. This was done to compare the therapeutic efficacy of one mAb against that of the combination of ch-mAbs.

Separate sets of mice were maintained for each experimental group infected with clade 1.0 for determination of viral titers and histopathology experiments. On day 3, 6 and 9 post viral challenges, mice were euthanized by a lethal dose of sodium pentobarbital. For determination of viral titers, lungs were aseptically removed. Tissues were homogenized in 1 ml Dulbecco's Minimal Essential Medium (DMEM) supplemented with antibiotic-antimycotic solution (Gibco-BRL, USA) to achieve 10-fold serially diluted suspensions of lung samples and were titrated on monolayers of MDCK cells. The viral titers were calculated by use of the method of Reed and Muench method [27] and expressed as log_10_ TCID_50_/gram of tissue ±S.E. The limit of virus detection was 1.5 log10 TCID50/gram of lung tissue specimen. For histopathology, mice were necropsied and the lungs were stored in 10% (wt/vol) neutral buffered formalin and embedded in paraffin and sectioned. Sections were stained with hematoxylin and eosin (H/E) prior to light microscopy examination and were evaluated for lung pathology.

## Results

### Selection of a pair of complementing monoclonal antibodies

A panel of seven neutralizing mAbs against influenza hemagglutinin (HA) was screened for high hemagglutination inhibition titers against different clades of H5N1 viruses. Based on the results of the HI assay, mAbs 2D9 and 4C2 were chosen for further studies due to their high HI activity (data not shown) against a wide range of rescued reassortant viruses from different clades. Both mAbs were found to be of the IgG1 isotype. The amino acids involved in forming the epitopes of the mAbs were analyzed using selection of neutralization escape mutants. Sequencing of the complete HA gene isolated from multiple escape variants to mAb2D9 carried single point mutations at amino acid positions 189 (Arg to Trp) or 223 (Ser to Arg) (excluding signal peptide). Similar analysis for mAb 4C2 revealed the involvement of amino acid 155 (Ser to Asn) in forming the epitope.

The two mAbs were found to recognize non-overlapping epitopes and reacted with all the H5N1 viruses from different clades available in our laboratory. Further, escape mutants to mAb 2D9 were recognized by mAb 4C2 and vice versa. Hence, these mAbs were thought to have good potential for being used in combination as therapy against H5N1 infections. To further ascertain this, the mAbs in combination were subjected to hemagglutination inhibition assays against a wide range of H5N1 viruses from different clades. Hemagglutination inhibition assays using a combination of these mAbs elicited a titer of 128–512 with all the tested H5N1 strains (Table 2). It was then concluded that the mAbs 2D9 and 4C2 complemented each other and were a good pair to use in therapy against H5N1 influenza.

**Table 2:** Hemagglutination Inhibition (HI) titers of the mAbs against H5N1 influenza viruses.

H5N1 strain	Clade	HI titers of the mAbs		
		2D9^a^	4C2^a^	2D9+4C2^b^
A/Hongkong/156/97	0	128	256	256
A/HongKong/213/03	1	512	256	512
A/Vietnam/1203/04	1	512	512	512
A/Indonesia/TLL014/06	2.1	128	256	256
A/Indonesia/CDC540/06	2.1	256	<8	128
A/Indonesia/CDC669/06	2.1	512	256	512
A/Indonesia/CDC1031/07	2.1	256	512	512
A/turkey/Turkey1/05	2.2	256	128	256
A/barheaded goose/Qinghai/12/05	2.2	256	128	256
A/Nigeria/6e/07	2.2	128	64	128
A/Egypt/0636-NAMRU3/07	2.2	64	256	128
A/Anhui/1/05	2.3	512	256	512
A/chicken/Nongkhai/NIAH400802/07	2.3	256	128	256
A/VietNam/HN31242/07	2.3	512	128	256
A/goose/Guiyang/337/06	4	128	256	256
A/chicken/Shanxi/2/06	7	256	64	128
A/chicken/Henan/12/04	8	256	256	512
EM* 2D9 mAb A/Indonesia/CDC669/06		<8	256	128
EM* 4C2 mAb A/Indonesia/CDC669/06		512	<8	256

a Concentration of mAb at 500 µg/ml.

b Concentration of each mAb at 250 µg/ml.

* EM indicates Escape Mutant against the mAb mentioned.

Chimeric monoclonal antibodies (ch-mAbs) were generated for both the mAbs such that the constant regions were replaced with those from human origin but variable regions remained from murine origin. The chimeric mAbs generated in this way were 66.6% humanized. The chimeric antibodies still retained the original properties of the murine mAbs (results not shown). The *in vitro* microneutralization titers dropped a little compared to the murine mAbs but still retained significant viral neutralization activity (Table 3).

**Table 3:** Microneutralization titers of the murine and chimeric mAbs against H5N1 influenza viruses.

H5N1 strain^a^	Clade	Microneutralization titers of the mAbs	
		2D9+4C2 Murine mAbs^b^	2D9+4C2 ch-mAbs^b^
A/HongKong/213/03	1	640	320
A/Indonesia/CDC594/06	2.1	320	320
A/Anhui/1/05	2.3	320	160
A/goose/Guiyang/337/06	4	320	320
A/chicken/Henan/12/04	8	320	160

a 100 TCID_50_ of each virus strain used for microneutralization assay.

b Concentration of each mAb at 250 µg/ml.

### Prophylactic potential of the combination of chimeric mAbs

**Figure 1:**
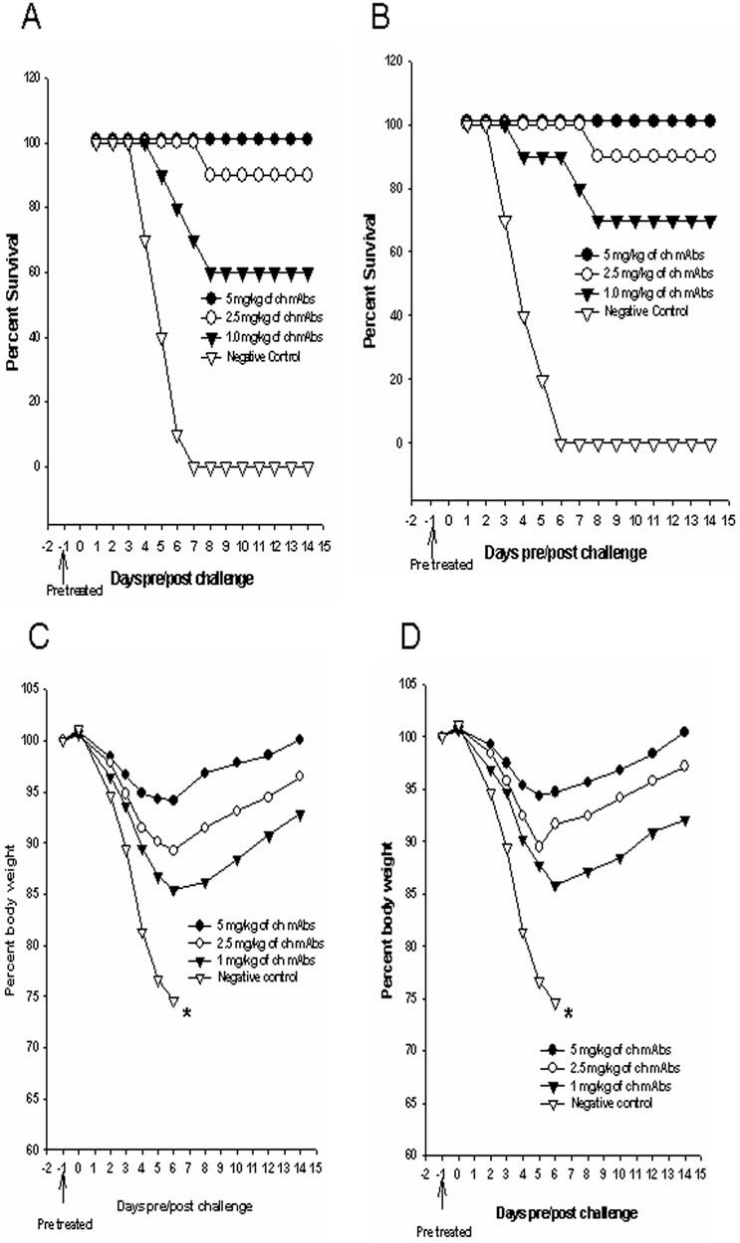
Prophylactic efficacy of the combination of chimeric mAbs in mice. Groups of mice (n = 10) were pre-treated intraperitoneally with 1 mg/kg, 2.5 mg/kg, 5 mg/kg or 0 mg/kg (PBS) of the combination of ch-mAbs, one day before challenge with 10MLD_50_ of mouse-adapted HPAI H5N1 from A/Vietnam/1203/2004 (A and C) or clade 2.1 virus A/TLL013/06 (B and D). An irrelevant IgG1 monoclonal antibody (specific for porcine circovirus) was used as a negative control. Mice were monitored for survival (A and B) and weight loss (C and D) throughout a 14 day observation period. The results are expressed in terms of percent survival and percent body weight (at the beginning of the trial) respectively.

The prophylactic efficacy of the combination of 2D9 and 4C2 ch-mAbs was evaluated against challenge with 10 MLD50 of clade 1 or clade 2.1 viruses. Groups of mice (n = 10) were inoculated via intraperitoneal route with different concentrations (1 mg/kg, 2.5 mg/kg and 5 mg/kg) of the combination of mAbs, 24 h prior to viral challenge. The negative control group of mice (treated with non-specific mAbs) showed the most rapid decline in body weight (above 25%) and died from complications associated with infection by day 6 post challenge. The group of mice which was pre-treated with a single dose of 5 mg/kg ch-mAbs showed less than 7% (Fig. 1C and 1D) loss of body weight and this concentration provided 100% protection against 10 MLD50 of both clades of viruses (Fig. 1A and 1B). Moreover, ch-mAbs at 2.5 mg/kg provided sufficient protection (90%) in a dose dependent manner and this group of mice showed less than 12% loss of body weight. Even at a very low concentration of 1 mg/kg, the ch-mAbs could provide 60% and 70% protection against clade 1(Fig. 1A) and clade 2.1(Fig. 1B) viruses respectively. The mice in these groups showed a loss of body weight of up to 15% (Fig. 1C and 1D).

### Therapeutic potential of a single mAb against viral challenge

**Figure 2:**
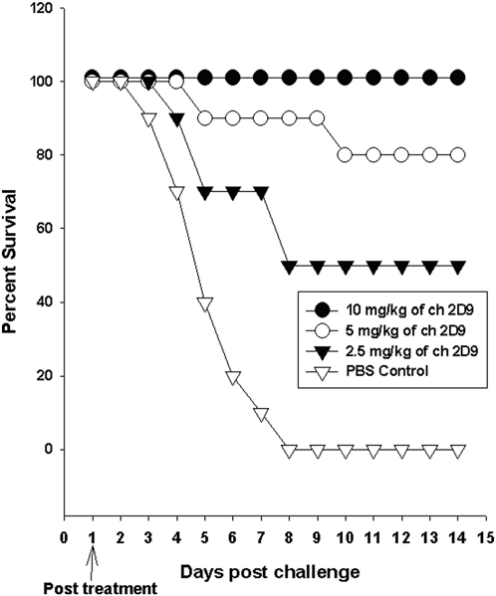
Therapeutic potential of a single mAb against H5N1 influenza infection in mice. Groups of mice (n = 10) were infected with mouse-adapted HPAI H5N1 from clade 2.1 virus A/TLL013/06. Twenty fours after viral challenge, the mice were treated via intra-peritoneal route with 2.5 mg/kg, 5 mg/kg, 10 mg/kg or 0 mg/kg (PBS) of a single ch-mAb 2D9. Mice were monitored for survival throughout a 14 day observation period. The results are expressed in terms of percent survival.

In order to evaluate the therapeutic potential of a single mAb against H5N1 influenza infection, we treated the mice with ch-mAb 2D9 alone, one day after viral challenge against 10MLD50 of H5N1 virus. We observed that 10 mg/kg of 2D9 ch-mAb (Fig. 2) could protect 100% of the mice from viral infection. However, only 80% and 50% of the mice could be protected with 5 mg/kg and 2.5 mg of therapeutic antibody respectively.

### Therapeutic potential of single dose versus two doses of the combination of chimeric mAbs

To determine if a single dose of treatment could elicit efficient protection against lethal viral infection, one set of mice were treated with a single dose (24 h after viral challenge) of mAbs. The efficacy of this single dose was compared with that in mice treated with double dose (24 h & 72 h after viral infection) of ch-mAbs.

**Figure 3:**
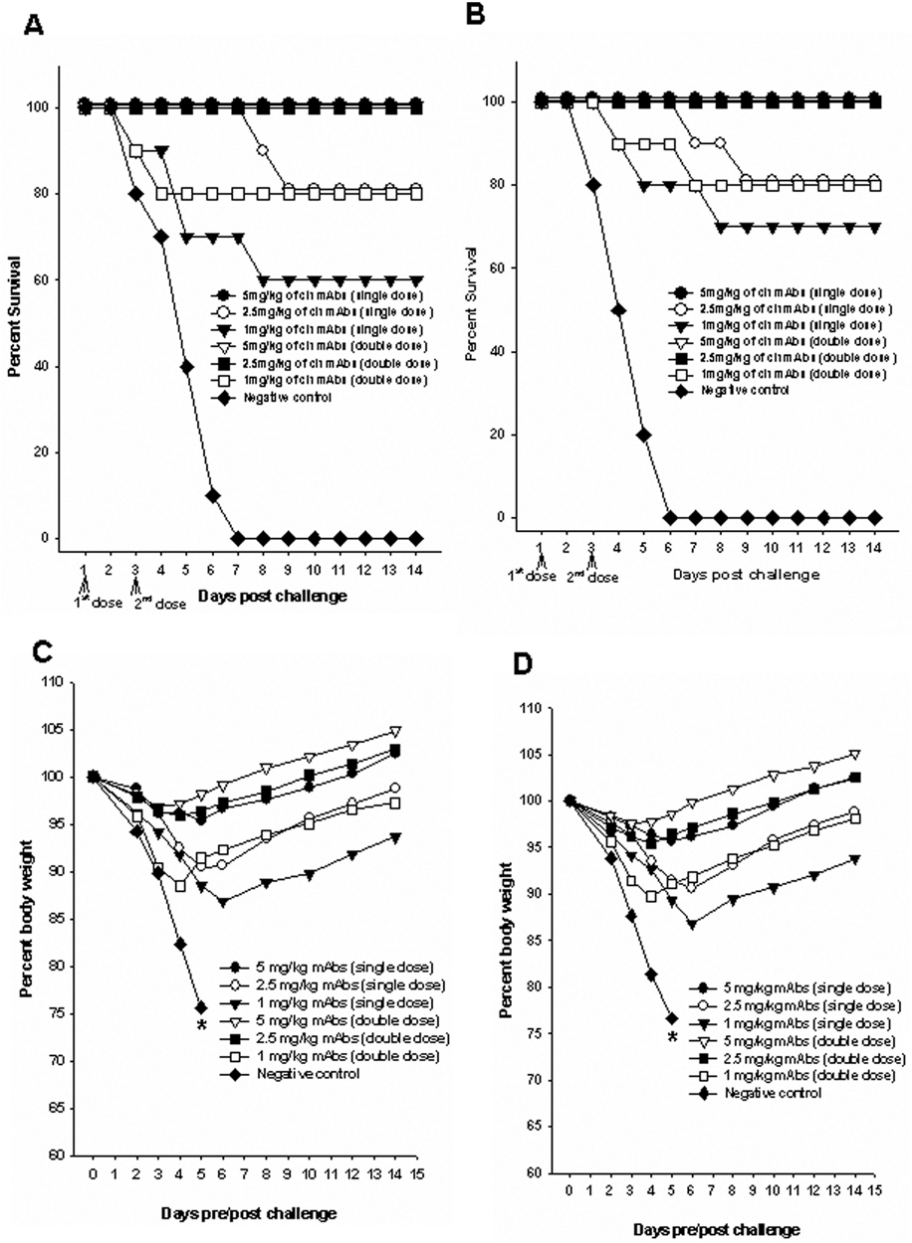
Therapeutic potential of one versus two doses of the combination of chimeric mAbs in mice. Groups of mice (n = 10) were infected with mouse-adapted HPAI H5N1 from Clade 1 A/Vietnam/1203/2004 (A and C) and clade 2.1 virus A/TLL013/06 (B and D). For treatment with a single dose, 24 h after viral challenge, the mice were treated via intra-peritoneal route with 1.0 mg/kg, 2.5 mg/kg, 5 mg/kg or 0 mg/kg (PBS) of the combination of mAbs. For treatment with two doses, different sets of mice were treated twice with similar doses of chimeric mAbs 24 h and 72 h after the viral challenge. An irrelevant IgG1 monoclonal antibody (specific for porcine circovirus) was used as a negative control. Mice were monitored for survival (A and B) and weight loss (C and D) throughout a 14 day observation period. The results are expressed in terms of percent survival and percent body weight (at the beginning of the trial) respectively.

Groups of mice treated with either single dose or double dose of 5 mg/kg ch-mAbs lost less than 5% of their original body weight by day 4 after challenge and provided 100% protection against both clade 1(Fig. 3A and 3C) and 2.1 viruses (Fig. 3B and 3D). Moreover, the group of mice that received the double dose of ch-mAbs (at 48 hour intervals) regained their body weight more rapidly (within 6 days) when compared to the mice that received a single dose, which regained their body weight only 10–12 days after the viral infection (Fig. 3C and 3D).

Mice treated with double dose of 2.5 mg/kg ch-mAbs also lost less than 5% of their original body weight and provided 100% protection against both H5N1 viruses. However, mice treated with a single dose of the same concentration of ch-mAbs showed considerable weight loss (up to 10%) and provided only 80% protection. Even at very low concentrations of 1 mg/kg, two doses of the combination of ch-mAbs could provide 80% protection against 10 MLD 50 of both H5N1 viruses. In contrast, mice treated with a single dose of the same concentration showed a loss in body weight of up to 15% (Fig. 3C and 3D) and provided only moderate protection (60–70%) against H5N1 viruses (Fig. 3A and 3B).

**Figure 4:**
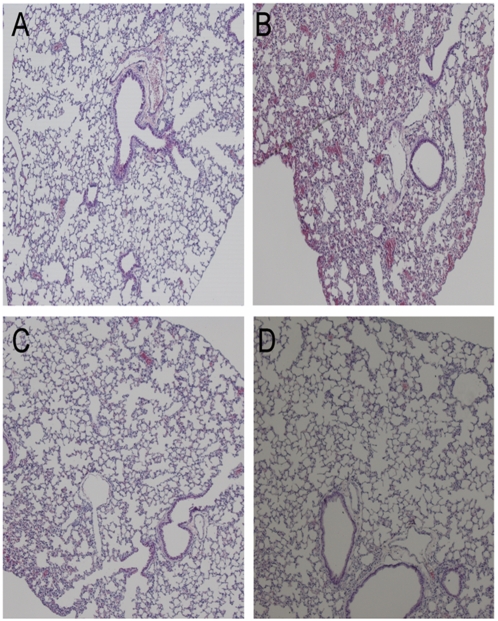
Histopathology of lung tissue in passively treated mice. Photomicrographs of hematoxylin and eosin stained lung sections of mice treated with single or double doses of the combination of mAbs after post experimental viral infection with Clade 1 A/Vietnam/1203/2004 H5N1 virus at 6 days post challenge. A) Normal morphology seen in uninfected mice, B) infected and untreated mice, C) mice treated with a single dose of 5 mg/kg of ch-mAbs at 24 h post infection, D) mice treated with two doses of 5 mg/kg of ch-mAbs at 24 h and 72 h post infection.

Histopathology studies were followed only for the lungs of mice treated with single and double doses of the combination of mAbs 24 h post viral infection with clade 1 virus. On day 6 p.i., lungs of untreated mice or mice treated with irrelevant mAb had pulmonary lesions consisting of moderate to severe necrotizing bronchitis, moderate to severe histiocytic alveolitis with associated pulmonary edema (Fig. 4B). The uninfected mice lacked lesions in the lungs (Fig. 4A). Mice treated with two doses of 5 mg/kg showed no lung pathology and looked similar to the uninfected control (Fig. 4D). Mice treated with a single dose of with 5 mg/kg of ch-mAbs had minimal bronchitis (Fig. 4C).

**Figure 5:**
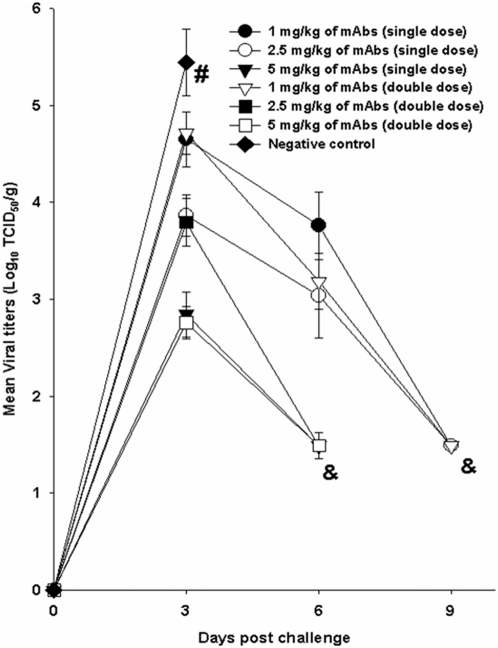
Measurement of viral infectivity titers in the lungs of mice experimentally infected with HPAI H5N1 (A/Vietnam/1203/2004- Clade 1) virus. For single dose treatment, 24 h after viral challenge, the mice were treated via intra-peritoneal route with 1.0 mg/kg, 2.5 mg/kg, 5 mg/kg or 0 mg/kg (PBS) of the combination of mAbs. For the double therapy experiment, different sets of mice were treated with similar doses of chimeric mAbs 24 h and 72 h after the viral challenge. The viral loads were measured in the lungs of the infected animals on days 3, 6 and 9 post challenge. The results are expressed in terms of mean value of log TCID50/g±(S.E). (# represents no survival of any animals in the group and & represents undetectable viral titers). The lower limit of detection was 1.5 log10 TCID50/g.

We studied the kinetics of viral replication by measuring the viral titers in the lungs of infected and treated mice on days 3, 6 and 9. The virus titers were most elevated on day 3 after viral challenge. Viral titers were highest in the infected but untreated control on day 3 and all the animals succumbed to the infection by day 6 after viral challenge (Fig. 5). The mice treated with a single dose of 5 mg/kg of the combination and those treated with double dose of 2.5 mg/kg or 5 mg/kg showed undetectable viral titers by day 6. However those mice treated with the other doses showed undetectable titers only by day 9 (Fig. 5). However on day 6, the mice treated with 2 doses of 1 mg/kg had much lower viral titers than those treated with single doses. These results show that even at lowest concentrations of 1 mg/kg, a double dose of the mAb combination could neutralize the virus much efficiently than a single dose (Fig. 5).

Escape mutants were isolated from the lungs of 20% of the mice after treatment with single dose of ch-mAbs, even at concentrations of 5 mg/kg. These Escape mutants were found to have a common mutation at Ser 155. Escape mutants were observed in 80% of mice when treated with 2.5 mg/kg of ch-mAbs. Among these, majority of the individual mice (75%) showed single point mutants that escaped the mAb and very few mice (25%) showed escape mutants with mutations at two points (Ser155 and Arg189). Interestingly, no escape mutants were isolated from the lungs of the groups of mice treated with double doses of ch-mAbs at 48 h intervals.

## Discussion

Highly neutralizing antibody responses for protective immunity against influenza infections have been associated with the hemagglutinin (HA) glycoprotein. Therefore, this protein has been a major focus for therapeutic intervention in influenza infections. Most of the influenza vaccines target this protein to induce immune responses in the host, mainly in the form of neutralizing antibody based response [25]. However, whenever immediate protection becomes essential and there is no time to induce an antibody response, the best alternative so far is passive immunization. Also, since drug resistant strains of H5N1 viruses are emergent, it is vital to explore other means of therapy for H5N1 infections [9]. Passive monoclonal antibody based therapy is a viable option that can be investigated.

Previous studies have proven the efficacy of humanized and human monoclonal antibodies as therapy in murine models of H5N1 infection [11], [12]. However, these studies only discuss the application of single monoclonal antibodies against infections with some strains of H5N1 virus. A single monoclonal antibody may not be sufficient to protect against all circulating strains [33]. Also, utilizing a single mAb against one epitope can result in selective pressure-induced “escape” of the virus through point mutations that can alter antibody binding [14]. The above mentioned studies with H5N1 infections have not looked into this aspect in great detail. In view of the proven efficiency of passive prophylaxis and therapy of H5N1 infection in animal models using single mAbs, we evaluated the prospect of using a combination of monoclonal antibodies to tackle the issues posed by using single mAbs as therapy. As is evident from our studies, the synergistic action of two or more mAbs in combination is required for preventing the generation of escape mutants and also to enhance the therapeutic efficacy against H5N1 infection.

We chose the mAbs based on their recognition of non-overlapping and non-competing epitopes. In combination, mAbs 2D9 and 4C2 could neutralize all of the strains from phylogenetically distinct clades 0, 1, 2.1, 2.2, 2.3, 4, 7 and 8. Also, the escape mutants generated from each of these mAbs could be efficiently neutralized by the other. We deduced that the combination of these mAbs would be very efficient in therapy against most strains of H5N1. Hence, we used the combination of these mAbs for prophylaxis and therapy against H5N1 in a mouse model of infection.

The results of the present study demonstrate that passive administration of a combination of two different neutralizing chimeric mAbs against HA1 can effectively protect against highly pathogenic H5N1 infection, when administered either as prophylaxis or therapeutics. We observed that 10 mg/kg of ch-mAb was required for the protection of 100% of the mice when a single mAb was used. However, when the combination of mAbs was used under the same conditions of viral challenge, only 5 mg/kg of a single dose or 2.5 mg/kg of the double dose was needed to offer complete protection. Administration of two doses of the combination showed better protection as the viral loads in the lungs were significantly reduced when compared to administration of a single dose. Moreover, a double dose of the combination of mAbs controlled immune escape as no escape mutants were isolated from the lungs of the groups of mice treated at 48 h intervals with two doses.

We observed the generation of escape mutants *in vivo* in 100% of the cases whenever a single monoclonal antibody was used for therapy (data not shown). However, using two monoclonal antibodies in combination also showed the generation of escape mutants whenever sub-neutralizing concentrations of mAbs were used. Using higher concentrations in a single dose (5 mg/kg) reduced this possibility. This may have been dependent on the amount of the complementary circulating mAb present in the system which could effectively neutralize the escape mutants due to any one of the mAb. Based on the evidence from the body weight of the mice, the mice were healthy enough from the single administration for the escape mutants to be cleared by the active immune system of the mice. Given that the half life of the mAbs is limited, it is evident as to why two doses of the mAb combination worked in a much better way and provided better safety against the emergence of escape variants. The high rate of emergence of escape mutants to these viruses are evidence that the antibodies are highly neutralizing and hence forcing the viruses to adapt. But the fact that using both ch-mAbs in combination in two doses did not give rise to any escape mutants is proof that the antibodies are complementary and hence offer complete protection to the mice.

Previous studies with other viruses have shown that combination of two or more than two mAbs directed against different epitopes could lead to a two to ten fold increase in neutralization titers [28], [29] and provided greater protection against many other diseases [30], [31], [32]. Moreover, Meulen et al. [33] reported that much better control of potential neutralization escape variants could be achieved with an antibody combination against SARS Coronavirus. However, no studies have been done so far to demonstrate the efficacy of combination therapy against H5N1 infection.

Though the dose of antibodies delivered for complete protection of mice was quite high, we believe that further improvement of these antibodies as well as their inclusion in an antibody cocktail will ensure better protection. Our data provide a rationale to develop combinations of mAbs for human H5N1 prophylaxis and therapeutics. The combination of two mAbs expanded the breadth of protection with a high level of efficacy and safety associated with potential immune escape variants. Also, combination therapy may allow for a lower dose of antibody to be administered for passive therapy of influenza infection and hence can be made available at reduced economic costs during an outbreak. In future, it may be possible to generate humanized monoclonal antibodies of mAb 2D9 and 4C2 by CDR (Complementarity Determining Regions) grafting and further facilitate their use in non-primate and human clinical trials.
